# Novel Optogenetic Approaches in Epilepsy Research

**DOI:** 10.3389/fnins.2019.00947

**Published:** 2019-09-06

**Authors:** Elvis Cela, Per Jesper Sjöström

**Affiliations:** ^1^Brain Repair and Integrative Neuroscience Program, Centre for Research in Neuroscience, Department of Medicine, Department of Neurology and Neurosurgery, The Research Institute of the McGill University Health Centre, Montreal General Hospital, Montreal, QC, Canada; ^2^Integrated Program in Neuroscience, McGill University, Montreal, QC, Canada

**Keywords:** epilepsy, optogenetics, seizures, animal models, plasticity, kindling

## Abstract

Epilepsy is a major neurological disorder characterized by repeated seizures afflicting 1% of the global population. The emergence of seizures is associated with several comorbidities and severely decreases the quality of life of patients. Unfortunately, around 30% of patients do not respond to first-line treatment using anti-seizure drugs (ASDs). Furthermore, it is still unclear how seizures arise in the healthy brain. Therefore, it is critical to have well developed models where a causal understanding of epilepsy can be investigated. While the development of seizures has been studied in several animal models, using chemical or electrical induction, deciphering the results of such studies has been difficult due to the uncertainty of the cell population being targeted as well as potential confounds such as brain damage from the procedure itself. Here we describe novel approaches using combinations of optical and genetic methods for studying epileptogenesis. These approaches can circumvent some shortcomings associated with the classical animal models and may thus increase the likelihood of developing new treatment options.

## Introduction

Epilepsy is a common neurological disorder affecting about 1% of the global population (Engel and Pedley, 2008; Brodie et al., 2009). It is defined as a chronic condition associated with at least one seizure and associated neurological, psychological, as well as cognitive consequences (Fisher et al., 2014). A seizure is defined as a transient occurrence of symptoms such as aberrant speech, perception, or attention thought to result from aberrant neuronal activity in the brain (Fisher et al., 2005).

Seizures may originate in different brain areas and have varying symptoms associated with the area of initiation (Brodie et al., 2009; Kwan et al., 2011). Once seizures arise, they are more likely to reoccur, giving rise to the notion that “seizures beget seizures” (Ben-Ari et al., 2008). There are, however, different types of seizures. Broadly, convulsive seizures are associated with body movements while non-convulsive seizures such as absence seizures are not. Epilepsy can also be crudely categorized into generalized or focal epilepsies, even though epilepsies that start as focal may eventually generalize (Engel, 2005).

Seizure types can also be divided into those that arise from genetic predispositions and those that are acquired. Genetic predispositions for developing seizures can for example arise as a result of channelopathies, which are each associated with specific seizure types and probabilities of having certain symptoms (Chang and Lowenstein, 2003; Glasscock et al., 2007). Other examples include mutations in transcriptional regulators such as JAK-STAT (Grabenstatter et al., 2014), proteins involved in cell growth such as mTOR (Way et al., 2012), or expansions in non-coding regions (Ishiura et al., 2018). On the other hand, acquired epilepsy can arise for many different reasons including: traumatic brain injury, infection, cancer, cerebrovascular disorder, autoimmune disorder, and developmental malformation (Berkovic et al., 2006).

Although traumatic brain injury accounts for 5% of epilepsies, typically a considerable period of time has to pass before the first seizures emerge (Herman, 2002). This latent period of epileptogenesis is thought to be due structural reorganization and network rewiring, although the details are poorly understood. For this reason, epileptogenesis is a central focus of animal studies in efforts to improve treatment outcomes (Chauvette et al., 2015).

Once epilepsy has been diagnosed, there are several options for treatment with two common avenues being surgical resection of diseased tissue and treatment with ASDs (Kwan et al., 2011). ASD treatment is typically prescribed but unfortunately approximately 30% of patients do not respond to this type of treatment (Kwan et al., 2011). Furthermore, patients taking ASDs may suffer from a plethora of side effects, such as drowsiness, dizziness, blurred vision and tremor (Perucca and Gilliam, 2012). Factors that contribute to a poor prognosis include presence of multiple seizure types, presence of generalized tonic-clonic seizures, and high seizure frequency before treatment (Kwan and Brodie, 2000; Kwan et al., 2011). Limitations of ASDs in managing seizures may arise from problems that plague many drugs, such as failure of optimal dosage and reliance on serum drug concentrations in lieu of monitoring clinical symptoms (Kwan et al., 2011). The lack of treatment specificity, along with the high percentage of ASD non-responders who as a consequence suffer a lower quality of life, necessitates a more advanced understanding of the basic mechanisms of epileptogenesis. Importantly, treating seizures with ASDs does not generally undo the process of epileptogenesis, as evidenced by the seizure remittance and relapse rates (Shorvon and Goodridge, 2013). This is not surprising, since epileptogenesis presumably involves structural and morphological changes that are unlikely to be reversed by ASDs. ASDs work best in suppressing generation, propagation and severity of seizures themselves (Chen Z. et al., 2018). Essentially, we need to better elucidate how seizures initially arise in the healthy brain and establishing better animal models is the key to this.

## Classical Animal Models of Epilepsy

A major thrust of epilepsy research has been to recapitulate seizures and their associated symptoms in animal models and study their progression over time. As a result, a variety of animal models have been developed to study different aspects of epilepsy. Two enduring groups of models used to study chronic epilepsy are genetic and acquired, further subdivided into electrical or chemical induction (Löscher, 2002). Genetic animal models can involve animals with spontaneous or induced mutations resulting in recurrent seizures. For example, mutations in GluA4 AMPARs have been causally linked to absence seizures (Paz et al., 2011). On the other hand, acquired electrical models, such as kindling, rely on gradual development of evoked seizures after repeated stimulation in initially healthy animals (Löscher, 2011). In chemical induction models, proconvulsants such as pentylenetrazol, kainic acid, or pilocarpine are injected into an animal to induce chronic seizures (Löscher, 2002). These chemicals act on receptors critically involved in synaptic transmission, thereby disturbing the excitatory-inhibitory balance and resulting in seizures (Fisher, 1989). On the other hand, 4-aminopyridine (4-AP) — another commonly used proconvulsant — acts mainly upon voltage-activated potassium channels, where it promotes elevated action potential firing due to faster recovery after inactivation (Storm, 1988), while kainic acid acts upon glutamate receptors (Olney et al., 1974). Several widely used convulsants act by antagonism of the GABA receptors (GABARs), thereby promoting excitability in the brain (Fisher, 1989). However, chemical kindling may also be elicited by agents such as cholinesterase agonists, opiates, local anesthetics, neurotoxicants, as well as excitatory amino acids (Stripling and Ellinwood, 1977; Cain, 1983; Mori and Wada, 1989; Gilbert, 1992).

Administration of chemical convulsants can be intracerebral or systemic. Chemical convulsants can be combined with electrical stimulation, or can be delivered alone repeatedly in a kindling paradigm (Cain, 1981). Finally, chemicals administered alone can cause *status epilepticus* if administered at high enough doses. For example, Clark et al. (1992) found that a single high-dose injection of cocaine (65 mg/kg) instantly caused seizures, whereas a lower dose (40 mg/kg) required multiple injections over several days to eventually elicit seizures.

What advantages does chemical kindling present over other animal model of epilepsy? First, chemoconvulsants such as kainic acid preferentially target the hippocampus, even when administered systemically, mimicking temporal lobe epilepsy (TLE) in humans (Nadler et al., 1978). Secondly, chemical kindling results in structural changes that resemble those in partial seizures, as seen with pilocarpine injections (Cavalheiro et al., 1991). Thirdly, chemoconvulsants such as PTZ or strychnine can be used as acute seizure models to screen ASD action without necessarily resulting in chronic epilepsy (Löscher, 2011).

There are also several drawbacks to using chemoconvulsants to study epilepsy. A main disadvantage is the lack of control over timing from chemoconvulsant injection to first seizure. Furthermore, variability in drug metabolism and dissemination from injection site adds uncertainty to the timing of the response and this is hard to control for. Finally, systemic injection of drugs can have off-target effects with unintended consequences secondary to seizure initiation. For example, kainic acid, which acts upon kainate receptors, has been used to model TLE seizures but the receptor distribution is not limited to the limbic structures (Li et al., 2001).

In electrical kindling models, healthy animals receive repeated electrical brain stimulation with what is an initially subconvulsive stimulus until it eventually elicits convulsions (Goddard et al., 1969). Seizures are thus not necessarily spontaneous, but are typically evoked by the kindling stimulus, although spontaneous seizures may also develop eventually (Pinel and Rovner, 1978; Michael et al., 1998). Repeated induction of evoked focal seizures through kindling is highly reliable. In addition, seizures are initially expressed at the stimulated site and generalize to other parts of the brain with repeated stimulation (Wada and Sato, 1974).

During the discovery of electrical kindling it was found that stimulation efficacy depends on the brain area stimulated, with the amygdala requiring the fewest number of stimulations (Goddard, 1967). The brain region-specific differences in response to kindling are thought to arise in part from differential reactivity in stimulation sites themselves as well as the areas that they connect to (Sato et al., 1990). The amygdala, for example, has strong connections with motor areas and is able to generate interictal spikes early during kindling, indicating that epileptogenesis may be taking place (Hopkins and Holstege, 1978; Racine et al., 1988). Kindling efficacy can be further enhanced by bilateral instead of unilateral stimulation (Kogure et al., 2000).

The early behavioral responses of freezing in response to stimulation typically progresses to generalized seizures with bilateral clonus (Racine, 1972). Correspondingly, the initial brief epileptiform activity that follows stimulation becomes altered, resulting in increased duration, amplitude, and frequency of seizures, typically also with a decreased seizure threshold (Racine, 1972). Also, kindled animals retain a reduced threshold for seizures over months, which was initially thought of as analogous to long-term memory (Goddard, 1967). Thus, kindling recapitulates in an animal model several key processes such as the evolution from partial to generalized seizures as well as structural and morphological changes that occur in human epilepsies (Racine, 1972; Sutula, 2004).

Because of the close links between kindling and information storage in the brain via synaptic learning (Sjöström et al., 2008), kindling opens up possible avenues for studying runaway plasticity as a key causative agent in epileptogenesis (Goddard, 1967). For example, kindling has similar induction requirements as LTP, it can be promoted by prior LTP as well as reversed by plasticity paradigms similar to LTD (Cain, 1989; McEachern and Shaw, 1999). Kindling is furthermore dependent on NMDA receptors, and epilepsy is known for hundreds of years to be treatable with marijuana (Piomelli, 2003; Alger, 2004; Sjöström et al., 2008). Further work has shown that CB1Rs may modulate NR2B-containing NMDAR internalization (Di Maio et al., 2019) and that they can interact with NMDARs to protect cells from excitotoxic insults (Liu et al., 2009; Sanchez-Blazquez et al., 2013). In keeping with a link to plasticity, both NMDA and cannabinoid receptor signaling are key to spike-timing dependent plasticity and to regulation of neurotransmitter release in neocortex (Sjöström et al., 2003; Abrahamsson et al., 2017).

However, NMDARs are not the only receptors tying kindling to plasticity; AMPARs have been investigated for both contributions to seizures as well as potential therapeutic targets (Rogawski, 2011; Rajasekaran et al., 2012). For example, seizures in early life alter calcium-permeable AMPAR expression, suggesting that they may promote aberrant long-term plasticity during kindling in synapse-type-specific manner (Rakhade et al., 2008; Larsen and Sjöström, 2015; Lalanne et al., 2016). There may also be a link between kindling-induced seizures and increased excitability of dendrites, which has been implicated in controlling plasticity (Bernard et al., 2004; Shah et al., 2004; Sjöström and Häusser, 2006; Sjöström et al., 2008).

What advantages does electrical kindling present over other animal models of epilepsy? First, electrical kindling allows precise focal activation of specific brain sites through anatomical targeting with the stimulating electrode. Second, it allows for reliable development of chronic seizures, as well as readily manipulated ictal and postictal periods. Lastly, behavioral alterations during stimulations start focally and evolve into generalized seizures, mimicking partial to complex seizure evolution in humans (Sato et al., 1990).

However, there are also several limitations to electrical kindling models. For example, using electrical kindling to study chronic epilepsy can be labor and time-intensive. Kindling typically also fails to capture a critical feature of epilepsy — recurrent spontaneous seizures — unless the animal is “over-kindled” through a large number of sessions (Pinel and Rovner, 1978; Michael et al., 1998). Since recurrent spontaneous seizures are a defining feature of epilepsy, this means kindling is not always identical to epilepsy, even though it may be useful as a model of key aspects of epilepsy, such as seizures. Finally, kindling is associated with injury and inflammation, which has been linked to higher seizure rates, making the contribution of pathological activity hard to distinguish from that of injury (Cavazos et al., 1994; Pitkänen et al., 2009).

To sum up, both electrical and chemical induction models reproduce many of the steps in seizure progression and related pathology underlying human epilepsies. However, because the perturbed cell population is unclear and because of the tissue trauma associated with the induction procedure, new approaches to study seizures are needed, e.g. using cell-specific targeting and activity manipulation. As we shall see below, optogenetics provides an indispensable tool here.

## Novel Genetic and Optical Approaches to Epilepsy

### Methods for Light-Driven Perturbation of Neuronal Activity

The cellular basis of seizure formation continues to evade researchers, in part owing to the unknown cell populations that electrical kindling recruits. Experiments combining brain imaging methods, such as fMRI, with epilepsy animal models may be able to resolve brain areas participating in seizures but cannot resolve specific cell populations (Lenkov et al., 2013; Gottschalk et al., 2016). Therefore, a causal understanding of epileptogenesis necessitates genetic identification and manipulation of activity in specific target neurons. Optogenetics can circumvent these drawbacks by allowing genetic tagging and manipulation of activity in specified neuronal populations (Zemelman et al., 2002). This technique allows light-driven activation or inactivation of neurons, by the expression of light-gated ion channels such as Channelrhodopsin-2 (ChR2) or pumps such as Halorhodopsin (NpHR), respectively (Nagel et al., 2002; Boyden et al., 2005; Lima and Miesenböck, 2005; Chow et al., 2010; Yizhar et al., 2011). The rhodopsin later termed ChR2 was discovered in the algae *Chlamydomonas reinhardtii*, where it generates photocurrents contributing to phototaxis (Harz and Hegemann, 1991). Aside from NpHR, another widely used inhibitory opsin is Archaerhodopsin (Arch) which inhibits neuronal activity through influx of H^+^ compared to NpHR’s extrusion of Cl^–^ ions (Nagel et al., 2002; Zhang et al., 2007; Chow et al., 2010).

Along with the first developed opsins used to activate and inhibit neurons, other variants include but are not limited to: G-protein-coupled receptors (opto-XRs) (Airan et al., 2009), vSWO/vLWO (Masseck et al., 2014), chloride channels (iC1C2, SwiChR) (Berndt et al., 2014) and chloride channel variants (JAWS) (Chuong et al., 2014), as well as sodium pumps (Inoue et al., 2013). Another approach has been to create a light-gated version of the ionotropic glutamate receptor (iGluR), which shares the advantage of millisecond manipulation of neuronal activity with optogenetics (Volgraf et al., 2006). Finally, reactive oxygen species can be used to inhibit neurotransmission after coupling to a light absorption by light-oxygen voltage domain (Shu et al., 2011). In summary, light-activated proteins open up novel approaches to studying epilepsy by permitting control of genetically defined neuronal populations, typically with millisecond precision.

### Genetic Targeting of Opsins

One key advantage of optogenetics is modulation of activity in specific neurons, but how can opsins be targeted to different neuronal populations? First, opsins can be delivered using adeno-associated virus (AAV) or lentivirus containing a promoter targeting the cells of interest (Ponnazhagan et al., 1997; Dittgen et al., 2004). Furthermore, the serotype of the AAV itself can confer a degree of selectivity though specific tropism (Burger et al., 2004). Secondly, opsins can be expressed in transgenic animals using a combination of the Cre/loxP (Sauer, 1987) and/or Flp/Frt systems (Buchholz et al., 1998), which can add even more specificity through selective promoters (Fitzsimons et al., 2002). Finally, specific cell populations can be targeted using monosynaptic and/or transsynaptic tracing techniques. Retrograde tracing can be done using rabies virus (Rancz et al., 2011; Chatterjee et al., 2018) or wheat germ agglutinin (Libbrecht et al., 2017) in combination with Cre-dependent ChR2 expression (Gradinaru et al., 2010). Anterograde tracing can be done using Herpes simplex virus (Lo and Anderson, 2011) or certain AAV serotypes (Zingg et al., 2017). These techniques can also be combined to improve spatial and/or temporal selectivity, by employing intersectional targeting strategies. For example, Cre or Flp can be fused to an estrogen receptor (ER) ligand-binding domain (LBD) that is only sensitive to synthetic 4-hydroxytamoxifen (4-OHT). Upon 4-OHT administration, the ER-LBD translocated to the nucleus where it can mediate recombination (Dymecki and Kim, 2007; Sjulson et al., 2016). Thus, recombination can be temporally regulated by 4-OHT injection.

### Putting a Brake on Seizures With Optogenetics

**FIGURE 1:**
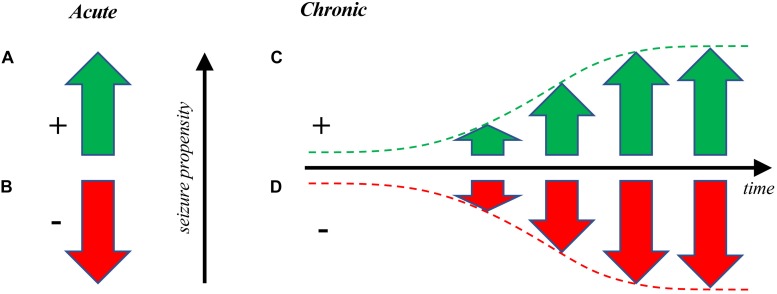
Optogenetic manipulation of seizure propensity. **(A)** Optogenetics can be used to drive seizures acutely (Khoshkhoo et al., 2017) or to **(B)** put a brake on already active seizures (Wykes et al., 2012; Krook-Magnuson et al., 2013) as a form of pro- or anti-convulsant, respectively. This latter approach has potential clinical applications for interrupting ongoing seizures (Paz et al., 2013), whereas both are useful for studying epilepsy. **(C)** Seizures can also be gradually elicited over time via optogenetic kindling, or optokindling (Cela et al., 2019). Optokindling is fundamentally distinct from directly driving seizures by optogenetic stimulation, since optokindling requires long-lasting changes of neuronal circuits (Cela et al., 2019), whereas direct optogenetic drive does not (Khoshkhoo et al., 2017). **(D)** It may also be possible to gradually decrease seizure propensity via optogenetic dekindling. As far as we know, this remains to be experimentally demonstrated optogenetically, but such findings have been reported with electrical stimulation (Bains et al., 1999; Ozen and Teskey, 2009). The kindling and dekindling modes may both enable the study of epileptogenesis, e.g. to test therapies that slow down or reverse the development of seizures.

Optogenetics has found multiple uses in neuroscience, including studying epilepsy where it has been used to both halt and initiate seizures (Table 1; Figure 1; Paz et al., 2013; Cela et al., 2019). The majority of studies attempting to optogenetically abate seizures have used classical animal models to induce epilepsy (Wykes et al., 2012; Wang et al., 2017).

**TABLE 1:** Optogenetic epilepsy studies.

**References**	**Brake**	**Drive**	**Kindle**	**Area**	
Cela et al., 2019			✓	Motor	Cortex
Khoshkhoo et al., 2017		✓		Motor	
Wykes et al., 2012	✓			Motor	
Assaf and Schiller, 2016	✓	✓		Somatosensory	
Chang et al., 2018		✓		Somatosensory	

Berglind et al., 2014	✓			CA3	Hippocampus
Berglind et al., 2018	✓			CA3	
Bui et al., 2018	✓			Dentate gyrus	
Chen S. et al., 2018	✓			CA1 + DG	
Chiang et al., 2014	✓			CA3	
Krook-Magnuson et al., 2013	✓			CA1	
Krook-Magnuson et al., 2015	✓	✓		Dentate gyrus	
Ladas et al., 2015	✓			Dentate gyrus	
Lu et al., 2016	✓			Dentate gyrus	
Osawa et al., 2013		✓		CA3 + DG	
Sessolo et al., 2015		✓		Temporal cortex	
Shiri et al., 2015		✓		Entorhinal cortex	
Tonnesen et al., 2009	✓			Hippocampus	
Wang et al., 2017	✓			Subiculum	
Weitz et al., 2015		✓		Hippocampus	
Yekhlef et al., 2015	✓			Entorhinal cortex	

Krook-Magnuson et al., 2014	✓			Lateral	Cerebellum
Kros et al., 2015	✓			LCN and MCN	

Soper et al., 2016	✓			Superior colliculus	Midbrain

Paz et al., 2013		✓		Ventrobasal	Thalamus
Sorokin et al., 2017	✓	✓		Ventrobasal	

*Studies to date using optogenetics either to interrupt (“Brake”), promote seizures (“Drive”) or kindle (“Kindle”) (also see Figure 1). DG denotes the Dentate gyrus, CA1 *Cornu Ammonis* subfield 1, CA3 *Cornu Ammonis* subfield 3, LCN lateral cerebellar nuclei, and MCN medial cerebellar nuclei.*

Interneurons (INs) play a central role in many types of epilepsy and different populations of INs have been critically linked to both the initial seizure activity (Khoshkhoo et al., 2017) and eventual generalization of seizures (Wang et al., 2017). In a handful of pioneering studies, it was shown that spontaneous seizures in a TLE induction model can be controlled by either stimulation of GABAergic cells or direct optogenetic inhibition of PCs in the hippocampus (Krook-Magnuson et al., 2013; Ladas et al., 2015; Lu et al., 2016). Inhibiting excitatory PCs using NpHR to stop or alleviate seizures has also been shown in hippocampus (Tonnesen et al., 2009; Sukhotinsky et al., 2013; Berglind et al., 2014; Krook-Magnuson et al., 2015). Finally, it was found that stroke-induced seizures can be stopped using optogenetic inhibition of thalamocortical neurons via NpHR (Paz et al., 2013).

However, inhibition is not always local. For example, it has been shown that TLE seizures can be inhibited by optogenetically stimulating the cerebellum (Krook-Magnuson et al., 2014). TLE seizure generalization is modulated differently by mossy and granule cells and these seizures are correlated with decreased spatial discrimination performance, mirroring similar deficits in human epilepsies (Bui et al., 2018). In another study, researchers used inhibitory opsins to suppress PC activity and abort seizures in a tetanus-toxin-induced model of neocortical epilepsy (Wykes et al., 2012). High-frequency light stimulation was both shown to inhibit 4-AP-induced seizures *in vivo* and *in vitro* (Chiang et al., 2014). Finally, seizure suppression has also been achieved in dorsal raphe and superior colliculus, highlighting the utility of optogenetics across several brain areas (Soper et al., 2016; Zhang H. H. et al., 2018).

Optogenetics has also been used to modulate seizures arising in genetic models where animals have a predilection to developing seizures. In absence epilepsy models, both activation of tonic spiking in thalamus and optogenetic stimulation of cerebellar neurons abated seizures *in vivo* (Kros et al., 2015; Sorokin et al., 2017). Taken together, these studies highlight different ways optogenetics can be used for seizure cessation, to potentially enable therapeutic treatments.

### Promoting Seizures With Optogenetics

Optogenetics has also been used to study the initiation of seizures and contributions of different cell types to seizure activity (Figure 1). Several studies have been able to induce seizures using only light stimulation. For example, stimulation of ChR2-expressing cells in the hippocampus led to seizure-like events (Osawa et al., 2013). Optogenetic stimulation of hippocampal PCs can also give rise to progressively intense seizures (Berglind et al., 2018). High-frequency optogenetic stimulation of PCs in dorsal or intermediate hippocampus give rise to different behaviors such as face twitching or clonus providing an explanation for the heterogeneity of seizure types seen in patients with hippocampal pathology (Weitz et al., 2015). Another study found kindling-like effects after optogenetic stimulation of dentate gyrus, in the form of increased in duration and severity of seizure responses (Krook-Magnuson et al., 2015).

PCs are not the only neurons participating in seizure activity, INs have been also implicated on different aspects of seizure formation. For example, activation of PV INs can induce seizures during the inter-ictal period but inhibit them during the ictal period (Assaf and Schiller, 2016). Further, optogenetic induction of seizures in neocortex revealed different roles of distinct IN populations with VIP inhibition increasing seizure threshold and somatostatin (SOM) and parvalbumin (PV) prolonging seizures during ictal activity (Khoshkhoo et al., 2017). Indeed, INs may play a role in the transition to ictal events through rebound excitation (Chang et al., 2018). Thus, seizure onset is not determined solely by PCs; it is increasingly recognized that certain IN populations such as VIP play a key role (Khoshkhoo et al., 2017).

Promoting seizures with optogenetics does not have to rely on light stimulation alone. Indeed, several studies took advantage of the established electrical kindling protocol and used it in parallel with light stimulation to investigate epileptogenesis (Wang et al., 2017; Berglind et al., 2018). Using light stimulation alone eliminates the amount of damage caused by the stimulating electrode during electrical kindling and allows optogenetic targeting of diverse cell populations to study the breadth of epilepsies occurring.

While optogenetic stimulation alone has been proven sufficient in several cases (Osawa et al., 2013; Khoshkhoo et al., 2017; Cela et al., 2019), many studies use a genetic model or chemical proconvulsants in conjunction with optogenetics. For example, light stimulation of somatosensory cortex of mice with genetic mutations increasing susceptibility to absence epilepsy also initiates seizure events (Wagner et al., 2015). In addition, electrical kindling can be combined with optogenetic stimulation to give rise to progressively worsening generalized seizures (Wang et al., 2017). Another study exploring the graded development of seizures in hippocampus following repeated light stimulation found progressively worsening and eventual spread of seizure activity (Berglind et al., 2018). These studies showed that optogenetic stimulation is sufficient to induce seizures, but more work remains to be done using long-term stimulation as well as monitoring additional seizure properties in awake behaving animals.

Furthermore, several recent studies using optogenetics to investigate the contribution of different neuronal population to seizures have reported conflicting results. In CA3 for example, optogenetic activation of PV INs led to excitatory GABA transmission and a boost of seizure activity (Ellender et al., 2014). In apparent contradiction, activation of GABA INs in the subiculum delayed generalized seizures after TLE (Wang et al., 2017). In addition, optogenetic PV IN activation leads to ictal discharges *in vitro* (Shiri et al., 2015) as well as during 4-AP application (Yekhlef et al., 2015). However, a previous study using ChR2 to activate PV cells near the seizure focus led to cessation of seizures (Sessolo et al., 2015). During ongoing ictal activity, raised intraneuronal chloride levels may contribute to depolarizing GABA responses, further promoting both spontaneous and optogenetically elicited seizures (Cohen et al., 2002; Timofeev et al., 2002; Staley, 2004; Magloire et al., 2019), an effect that depends on KCC2 (Silayeva et al., 2015; Magloire et al., 2019). These conflicting results relied on optogenetics to highlight the complexity of IN contribution to seizure dynamics and underscore the need for further study. Future studies would benefit from looking at other IN types such as VIP and SOM to investigate the relative contribution of different IN populations to seizure formation.

### Optogenetic Kindling Models of Epilepsy

Multiple studies have shown that seizures can be acutely elicited with optogenetics (Table 1 and Figure 1). In light of this, is there a place for using optokindling? We argue that there are several key advantages. Because optokindling allows graded development, increased severity of electrographic and behavioral seizures over time, as well as long-term susceptibility of seizure properties akin to long-term memory (Table 2; Goddard, 1967; Teskey, 2001), it is possible to use optokindling to study aspects of epileptogenesis rather than simply studying directly driven seizures. These features make kindling a reliable animal model for the study of the transition to seizures from a healthy brain over the long term (Sato et al., 1990).

**TABLE 2:** Hallmark features of kindling.

	**Metric**	**Description**
1	Electrogenic seizure severity	Kindling causes an increase in duration, amplitude, frequency, and complexity of seizures.
2	Behavioral seizure severity	Behavioral seizures appear and increase in severity.
3	Seizure threshold	Seizure threshold is reduced, so that initially inert stimulation may eventually elicit seizures.
4	Seizure propagation	Seizure eventually propagates from stimulation site to distant brain regions.

*Key properties of kindling, as outlined by Teskey (2001).*

Additionally, kindling can be combined with a second factor in a two-hit model of seizures to examine synergistic effects (Berkovic et al., 2006). For example, the two-hit model can be used to study how early-life seizures impinge on seizure propensity later in life (Koh et al., 2001).

**FIGURE 2:**
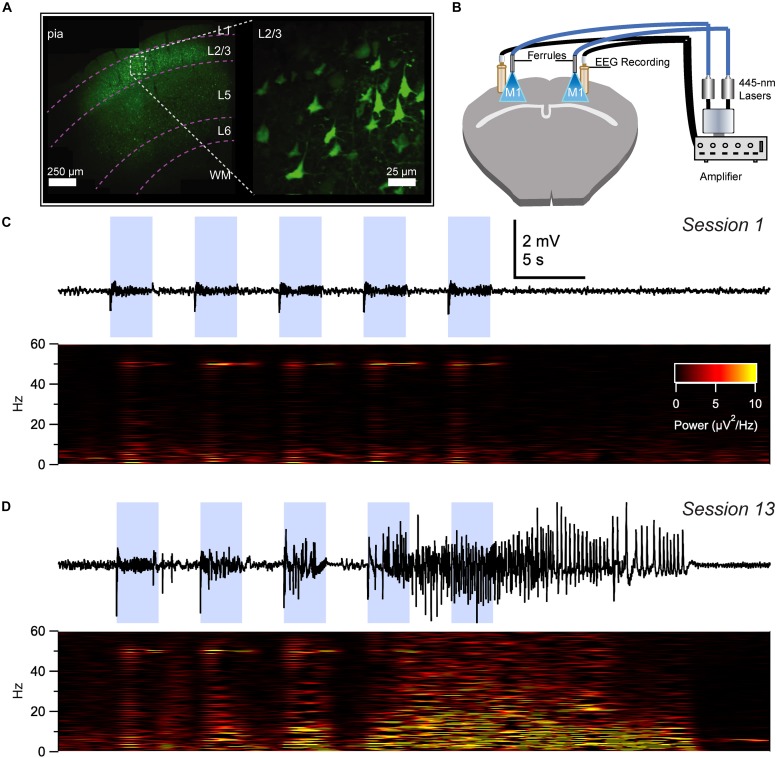
Optokindling via simultaneous EEG recording and ChR2 stimulation in awake behaving animals. **(A)** Coronal M1 section immunostained for EYFP indicated ChR2 expression in layer 2/3 (L2/3), 5, and 6, though predominantly in L2/3. Inset shows close-up of L2/3 ChR2-expressing PCs. **(B)** To simultaneously activate ChR2 and acquire EEG, ferrules and recording screws were implanted bilaterally above M1, without penetrating the cortex. Fiber optic cables were air-coupled to 445-nm lasers. EEG signals were processed by an extracellular amplifier, but not pre-amplified. A computer (not shown) TTL-gated the lasers and digitized amplified EEG signals. **(C)** Sample EEG trace illustrating that M1 optokindling did not elicit seizures in stimulation session 1 of 25 (Top). Spectrogram shows direct light-driven responses in the 50-Hz band but no seizures (Bottom). **(D)** M1 optokindling in session 13 elicited a prominent seizure in this sample EEG sweep (Top). Spectrogram reveals both light-driven responses in the 50-Hz band as well as increased power in low-frequency bands (Bottom). Reproduced and modified from Figure 1 in Cela et al. (2019), with permission.

With these advantages in mind, we recently developed an optokindling model in neocortex that replicated several hallmark features of electrical kindling (Table 2), including gradual seizure development as well as long-term seizure retention (Cela et al., 2019). With optokindling, we were able to induce seizures (Figure 2) whilst avoiding tissue damage (Cela et al., 2019), which might otherwise confound interpretation of results (Pitkänen et al., 2009). We found that optokindling could mimic the gradual development of seizures in epileptogenesis (Morrell, 1985), as an initially inert stimulus eventually elicited electrographic (Figure 2) as well as behavioral seizures (Cela et al., 2019).

Going forward, in order to effectively develop optogenetic variants to classical kindling, classical changes accompanying kindling must be measured such as long-term retention of seizure susceptibility, worsening electrographic and behavioral seizures, as well as changes in seizure threshold and possible propagation of seizures (Table 2; Goddard et al., 1969; Teskey, 2001). These variables are critical to differentiate optokindling models from direct optogenetic drive of seizures (also see Table 1).

Furthermore, optogenetics also opens up new avenues of examining dekindling, or the gradual decrease in seizure propensity, i.e. the opposite of epileptogenesis (Figure 1; Morrell, 1960; Salanova et al., 1996). With optogenetics, it may be possible to reverse the synaptic changes occurring during epileptogenesis. Future studies also need to examine other brain areas aside from neocortex to ensure that the optokindling effect can be replicated elsewhere in the brain as was done with electrical kindling (Goddard et al., 1969; Cela et al., 2019).

## Future Directions and Conclusion

Over several decades, the field of epilepsy has benefited from multiple animal models of epilepsy, as described above. More recently, optogenetics has been used to study epilepsy with the goal of linking circuit properties to seizures in defined neuronal populations. Here, we have discussed how optogenetics was used to both activate and inactivate target cells leading to either prolonging or reducing seizure duration in several different brain regions (Table 1).

However, several issues remain before optogenetics will likely gain widespread use in epilepsy research. First the virus injection and ferrule implantation procedure that is required for optogenetic activation is time consuming and labor intensive. This has already begun to be addressed by near infrared (IR) versions of ChR2 which do not need ferrule implants for light delivery and can instead be activated transcranially (Lin et al., 2013). These IR variants also reduce the possibility of heat-induced light damage from prolonged stimulation (Christie et al., 2013; Hososhima et al., 2015). Secondly, the EEG recording, which also requires time-consuming implantation procedures, can be replaced by all-optical interrogation of neural circuits using genetically encoded sensors for readout and optogenetic actuators for control of cellular activity (Emiliani et al., 2015). Thirdly, the advantage of optogenetics in targeting different neuronal populations is restricted by the specificity of factors such as promoters and viral serotype.

As the genetic toolkit for exogenous protein expression continues to expand (Sjulson et al., 2016), it will allow for the activity of neural circuits contributing to seizure formation to be recorded and manipulated by light, which in turn will enable the development of novel epilepsy models with improved spatiotemporal precision (Zhang Z. et al., 2018). This will likely be particularly useful in closed-loop systems where real-time adjustment can be made based on behavior or ongoing brain activity, e.g. at the start of seizures to shut them down.

Optokindling is a particular subset of this genetic toolkit — studies such as ours (Cela et al., 2019) will hopefully improve our understanding of circuit changes in epileptogenesis rather than of acute seizure induction. This is important since long-term seizure development may closely mimic certain aspects of human epilepsies that involve circuit alterations while acute seizures models may be more useful to study circuits and seizure properties as such (Sutula, 2004; Lillis et al., 2015).

Optogenetics is not the only genetic neuronal activation system useful for the study of epilepsy. Another system that has been used is pharmacogenetics using designer receptors exclusively activated by designer drugs (DREADDS). DREADDS are G-coupled receptors and can be used to inhibit (hMD4i receptor) or excite (hM3Dq receptor) cells (Pei et al., 2008) upon addition of the drug clozapine N-oxide (CNO). DREADDS have been used to both halt and initiate seizure development, e.g. seizure suppression in organotypic hippocampal slice cultures (Avaliani et al., 2016), seizure blockade in amygdala-kindled mice (Wicker and Forcelli, 2016), as well as silencing of pilocarpine-induced seizures *in vivo* (Katzel et al., 2014).

A recent study has highlighted the possibility of using optogenetics along with pharmacogenetics to induce seizures with the former while abating their activity with the latter (Berglind et al., 2018). DREADDS can be used to chronically inhibit or promote activity in genetically defined neurons similar to optogenetics but while DREADDS can serve some of the main properties of optogenetics in studying seizures, they have significant disadvantages. First, DREADDS have reduced temporal specificity compared to optogenetics. Chronic manipulation of activity levels imposed by DREADDS lack the millisecond-scale precision mimicking synaptic transmission afforded by optogenetics. Second, the supposedly otherwise inert agonist (CNO) can in fact be metabolized back to clozapine and have off-target effects such as lowered seizure threshold, which could of course cloud the interpretation in epilepsy studies (Sajatovic and Meltzer, 1996; Manvich et al., 2018). Optogenetics may thus prove to be a better method of activity manipulation when studying seizure initiation. However, when it comes to seizure control, DREADDS may be advantageous due to the steady modulation of excitability that they provide without the need for repeated intervention, as is the case for optogenetics.

In conjunction with genetic methods for manipulating neuronal activity, the roles of injury and inflammation can be explored using the two-hit model (Berkovic et al., 2006). Optical induction models such as optokindling have a narrower focus on activity-dependent plasticity, since — compared to their electric or chemical counterparts — they result in relatively little tissue damage or inflammation (Cela et al., 2019). It may in fact be possible to optokindle transgenic mice without the need for viral injection or surgery (Cela et al., 2019), thus bypassing injury and inflammation completely. This may make them especially useful for discovering novel therapies, to halt or slow down pathological plasticity in epileptogenesis.

The application of optogenetic intervention in human epilepsies has been delayed because several hurdles need to be overcome. For example, there are health concerns associated with using viral vectors for gene delivery. Furthermore, the human brain is much larger than that of rodents, which means much more powerful light sources are required to access deep brain structures (Couzin and Kaiser, 2005; Delbeke et al., 2017). Furthermore, optogenetic seizure interruption requires highly accurate real-time seizure detection, which is still a largely unsolved issue (Dheer et al., 2017). Nonetheless, there are several avenues for anti-epileptic optogenetic intervention in humans. For example, MRI-compatible fiber optrodes can be used to suppress seizures after injection of AAV-NpHR in epileptic tissue (Duffy et al., 2015).

Although it remains unknown what precisely the future holds in store, what should be clear from this review is that optogenetics is here to stay as a key tool to control activity in primary epilepsy research as well as in future treatments. Recent optogenetic studies have already revealed e.g. the differential IN population role in seizure initiation as well as different ways of halting seizures depending on the brain area (Paz et al., 2013; Ellender et al., 2014; Khoshkhoo et al., 2017). With optogenetics, additional circuit components underlying seizures will be dissected and novel therapeutic interventions will be discovered.

## Author Contributions

Both authors wrote the manuscript.

## Conflict of Interest Statement

The authors declare that the research was conducted in the absence of any commercial or financial relationships that could be construed as a potential conflict of interest.
